# Hydrogel Stack‐Tailored Logics and High‐Fidelity Multimodal Sensors Promoted by Precisely Evaluated Ionic Migration

**DOI:** 10.1002/advs.75848

**Published:** 2026-06-04

**Authors:** Haoran Chen, Hongjian Zhang, Zhonghui Shen, Taeuk Eom, Hyunseung Kim, Delong He, Jinbo Bai, Geon‐Tae Hwang, Yong Zhang, Chang Kyu Jeong

**Affiliations:** ^1^ State Key Laboratory of Advanced Glass Materials School of Materials Science and Engineering Wuhan University of Technology Wuhan China; ^2^ Center for Smart Materials and Device Integration Wuhan University of Technology Wuhan China; ^3^ Department of JBNU‐KIST Industry‐Academia Convergence Research Jeonbuk National University Jeonju Jeonbuk Republic of Korea; ^4^ Division of Advanced Materials Engineering Jeonbuk National University Jeonju Jeonbuk Republic of Korea; ^5^ Department of Energy Storage/Conversion Engineering of Graduate School (BK21 FOUR) Hydrogen and Fuel Cell Research Center Jeonbuk National University Jeonju Jeonbuk Republic of Korea; ^6^ LMPS‒Laboratoire De Mécanique Paris‐Saclay Université Paris‐Saclay CentraleSupélec, ENS Paris‐Saclay CNRS Gif‐sur‐Yvette France; ^7^ G.‐T. Hwang, Department of Materials Science and Engineering Pukyong National University Busan Republic of Korea

**Keywords:** energy harvesting, ionic diode, logic circuit, self‐powered, sensor array

## Abstract

Self‐powered flexible sensors represent indispensable components in tactile sensing and wearable electronic systems. In biological organisms, intracellular and extracellular ion transport underpin the precise perception, transmission, and processing of tactile stimuli. Inspired by these natural mechanisms, four types of self‐powered multifunctional sensors were developed based on the controlled motion of ions within cationic poly(diallyldimethylammonium chloride) and anionic sodium polystyrene sulfonate ionomers. The sensors exhibit a p–n junction configuration, where a depletion layer is established at the ionomer interface. Through the incorporation of 2D MXenes and 1D carbon nanotubes (CNTs), the electrical conductivity was optimized, yielding an open‐circuit voltage of approximately 75 mV and a short‐circuit current density of ∼67 µA cm^−^^2^. The distinct rectification behavior (ratio ≈ 8.8) enables logic circuit functionality, while the tunable assembly of sensing units into arrays allows precise discrimination of compression, bending, and directional stress stimuli. Unlike conventional pressure‐sensing arrays, each unit in the present system displays unique sensing characteristics. This work offers a new paradigm for the rational design of high‐performance, self‐powered ionic sensors for next‐generation flexible and wearable electronics.

## Graphical abstract

Inspired by biological ion transport, this work introduces self‐powered multifunctional sensors utilizing tailored hydrogel stacks. Optimizing ionic migration with MXenes and carbon nanotubes achieves high‐performance energy harvesting and unique logic circuit functionality. The resulting arrays precisely discriminate between compression, bending, and directional stress, offering a new paradigm for high‐fidelity wearable electronics.

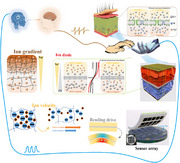

## 1 Introduction

Harvesting low‐frequency mechanical energy generated during daily human activities offers a promising approach for powering wearable and portable electronic devices [1, 2]. In addition, the accurate perception and analysis of tactile signals enable the interpretation of human actions, forming the fundamental basis for tactile sensors in human–machine interfaces [3, 4, 5]. Self‐powered tactile sensors, which simultaneously harvest mechanical energy, hold significant potential for applications in flexible and robotic electronics [6, 7, 8, 9]. Over the past several decades, extensive efforts have been devoted to developing piezoelectric and triboelectric energy harvesters to enhance electromechanical coupling efficiency for self‐powered sensing. However, their intrinsic limitations—particularly poor efficiency at low frequencies (<5 Hz) and extremely high impedance matching requirements—remain major challenges that restrict practical implementation [10, 11, 12, 13, 14].

By regulating the migration of mobile ions, several innovative strategies have been proposed for mechanical energy harvesting and high‐resolution tactile sensing, including ionic diode junctions, nanofluidic systems, and potentiometric transduction mechanisms [15, 16, 17, 18, 19]. Upon external mechanical stimulation, the expansion and contraction of soft solid matrices induce directional migration of charge carriers (mobile cations and/or anions), thereby generating measurable current signals [20, 21, 22, 23]. Taking advantage of the much slower mobility of ions than electrons [24], the response frequency of self‐powered sensors could be modulated and pulled down to the range of human motions (<5 Hz). The rapid evolution of soft robotics and electronic skins has highlighted the critical need for such low‐frequency, high‐sensitivity interfaces to enable dexterous manipulation and human‐motion monitoring [25, 26], positioning them as key components for next‐generation human‐machine interfaces and wearable electronics.

Recent advances in functional materials, particularly in MXene‐based composites, have significantly enhanced the mechanical and electrical performance of hydrogel sensors [27, 28, 29]. However, despite these material‐level breakthroughs, the functional architecture of most hydrogel sensors remains confined to conventional “spatial mapping” of pressure [30, 31, 32, 33]. To transcend this limitation, there is an urgent need to engineer “Perceptual Logic” at the device level.

Hydrogel‐based ionic diode devices, which emulate the operating principle of p–n junction diodes, have demonstrated a state‐of‐the‐art charge density of ≈4 mC cm^−^^2^ at 0.01 Hz, outperforming previously reported systems [34]. Although controlled ionic migration in ionotronic devices has yet to be fully realized, it plays a fundamental and critical role in modulating both energy harvesting performance and sensing capability, thereby expanding the scope of practical applications. Furthermore, the favorable rectification characteristics of ionic diodes enable the construction of logic circuits—an achievement unattainable in conventional piezoelectric and triboelectric energy harvesting systems [35, 36, 37, 38, 39, 40]. However, most existing ionic logic devices lack the capability for multimodal deformation recognition under complex stress states [41, 42]. The integration of high electromechanical coupling efficiency at low frequencies with inherent rectification functionality positions ionic diode‐based devices as highly promising candidates for advanced human–machine interfaces, soft robotics, and intelligent sensing platforms.

In this work, we design ionic diode‐based self‐powered pressure sensors utilizing anionic poly(diallyldimethylammonium chloride) and cationic sodium polystyrene sulfonate ionomers. A conductive network composed of 1D carbon nanotubes (CNTs) and 2D MXenes is integrated to facilitate ion and charge transport, thereby markedly enhancing the energy harvesting performance. The devices deliver a maximum power density of approximately 2.15 µW cm^−^^2^, surpassing most previously reported ionotronic energy‐harvesting systems. Four distinct sensing models are proposed based on variations in ionic migration behavior. This study represents the first demonstration of ionic diode junction‐based devices achieving high‐efficiency electromechanical coupling under both compressive and bending modes. Through precise engineering of four types of sensing units, a uniquely configured pressure‐sensing array is developed, capable of mapping and distinguishing spatially distributed compression and bending forces simultaneously. This capability for directional stress recognition represents a significant advancement in the field of self‐powered tactile sensing.

## 2 Results and Discussion

**FIGURE 1:**
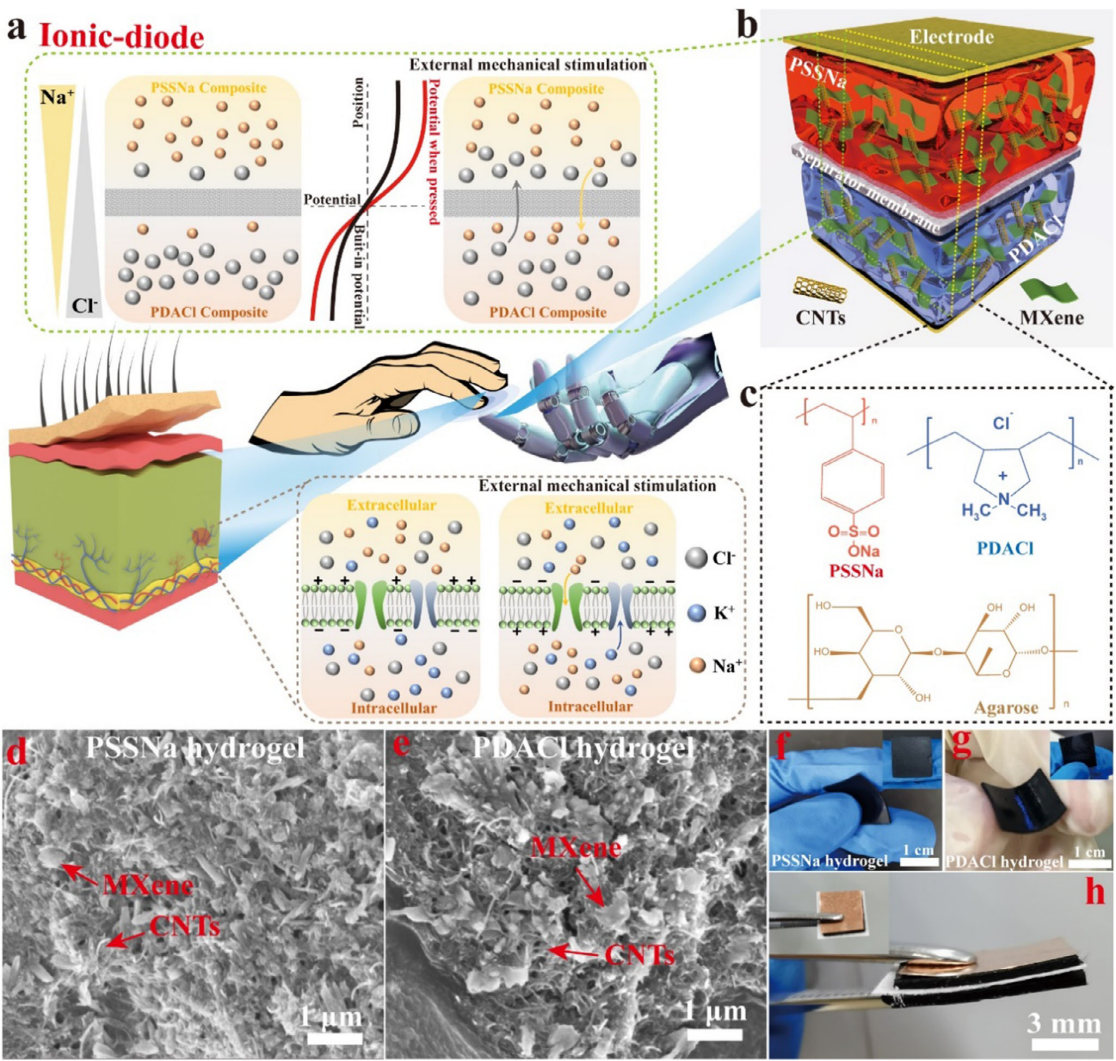
(a) Design concept of the ionic‐diode, inspired by the ion immigration process between intracellular and extracellular of human skin. (b) Schematic illustration of the designed hydrogel ionic‐diode. (c) Chemical structures of PDACl, PSSNa, and agarose. (d,e) SEM images and (f,g) Photographs of PSSNa/MXene/CNTs and PDACl/MXene/CNTs hydrogels. (h) Assembled hydrogel device.

Inspired by the ion migration processes occurring between intracellular and extracellular regions of human skin, we designed four types of ionic diode junction–based electromechanical coupling devices (Figure 1a; Figure S1). To establish a standardized nomenclature, components are defined as follows: cationic poly(sodium 4‐styrenesulfonate) (PSSNa) as Type A and anionic poly(diallyldimethylammonium chloride) (PDACl) as Type B. Here, subscripts 1 and 2 (e.g., A_1_/A_2_) denote hydrogels of the same type but with different concentrations, while 0 represents a pure agarose hydrogel matrix devoid of functional ionic pairs.

Based on these components, four distinct sensing models are proposed, each named to reflect its specific stacking configuration: (1) The A/B model; (2) The AB/0 model; (3) The A_1_/A_2_ model; (4) The A/A model. These configurations comprises a distinct stacking configuration of hydrogel matrices, capable of modulating ion migration under compressive or bending mechanical stimuli. Such deformation alters the local ion concentration through volume variation within the hydrogel, thereby generating a driving force for ionic transport. Each model operates through a unique mechanism: AB/0 functions via potential differences arising from unequal ion migration rates under a uniform concentration gradient; A_1_/A_2_ relies on an initial concentration gradient to drive ion motion; A/B is governed by the ionic diode effect; and A/A responds through asymmetric deformation–induced ion migration during bending. The rational integration of these four ionic diode sensor types into a pressure‐detection array enables discrimination between compression and bending modes, as well as identification of stress direction—representing the first demonstration of such multifunctional sensing capability in this field.

### 2.1 A/B Type Stack

Figure 1b illustrates the schematic configuration of the ionic diode, which consists of two distinct hydrogel layers— PSSNa and PDACl. Free Na^+^ and Cl^−^ ions can migrate across the depletion layer (Figure 1b), leaving the corresponding PSS^−^ and PDA^+^ polymer backbones behind. A depletion region with a thickness of several micrometers forms near the polytetrafluoroethylene (PTFE) separator membrane (pore size ≈ 1 µm). The two hydrogels are separated by this PTFE membrane to prevent electrical short‐circuiting, and copper electrodes are attached to both sides for electrical characterization. Agarose was chosen as the hydrogel matrix owing to two key attributes: (i) its ability to form a molecular network through interchain hydrogen bonding and retain large quantities of water via swelling, thereby facilitating efficient ion transport [43, 44]; and (ii) its excellent biocompatibility, which makes it a suitable candidate for human–machine interface and wearable electronic applications.

The uniform dispersion of 2D MXene nanosheets and 1D carbon nanotubes (CNTs) within PDACl/CNT/MXene and PSSNa/CNT/MXene composites provides efficient charge‐conduction pathways (Figures 1c,d), thereby enhancing the overall energy‐harvesting capability. Figure S2a,b presents the X‐ray diffraction (XRD) patterns of the MXene MAX phase before and after etching in an HCl/LiF mixed solution. The MAX phase, belonging to the *P6_3_/mmc* space group, exhibits hexagonal crystal symmetry [45]. After etching, incorporation of hydroxyl groups, fluoride ions, and water molecules causes a clear downward shift of the (002) reflection. Concurrently, the disappearance of the (104) peak indicates the complete removal of Al layers and the breaking of Ti─Al bonds. Figure S3a,b reveal the densely stacked multilayered Ti_3_C_2_T_x_ with a typical accordion‐like morphology, while TEM images (Figure S3c,d) show the transparent features of exfoliated single‐layer MXene sheets. Selected‐area electron diffraction (SAED) patterns confirm the preserved hexagonal carbide structure after exfoliation [46]. Atomic force microscopy (AFM) measurements (Figure S4) indicate a nanosheet thickness of approximately 1.5 nm, further confirming its single‐layer nature. Elemental mapping (Figure S5) demonstrates the homogeneous distribution of Ti and C elements within the hydrogel, verifying the uniform dispersion of conductive MXene nanofillers. Finally, the optical images of the hydrogel components and the fully assembled device (Figure 1f–h) highlight their excellent mechanical flexibility and structural integrity, establishing a solid foundation for long‐term operational durability.

**FIGURE 2:**
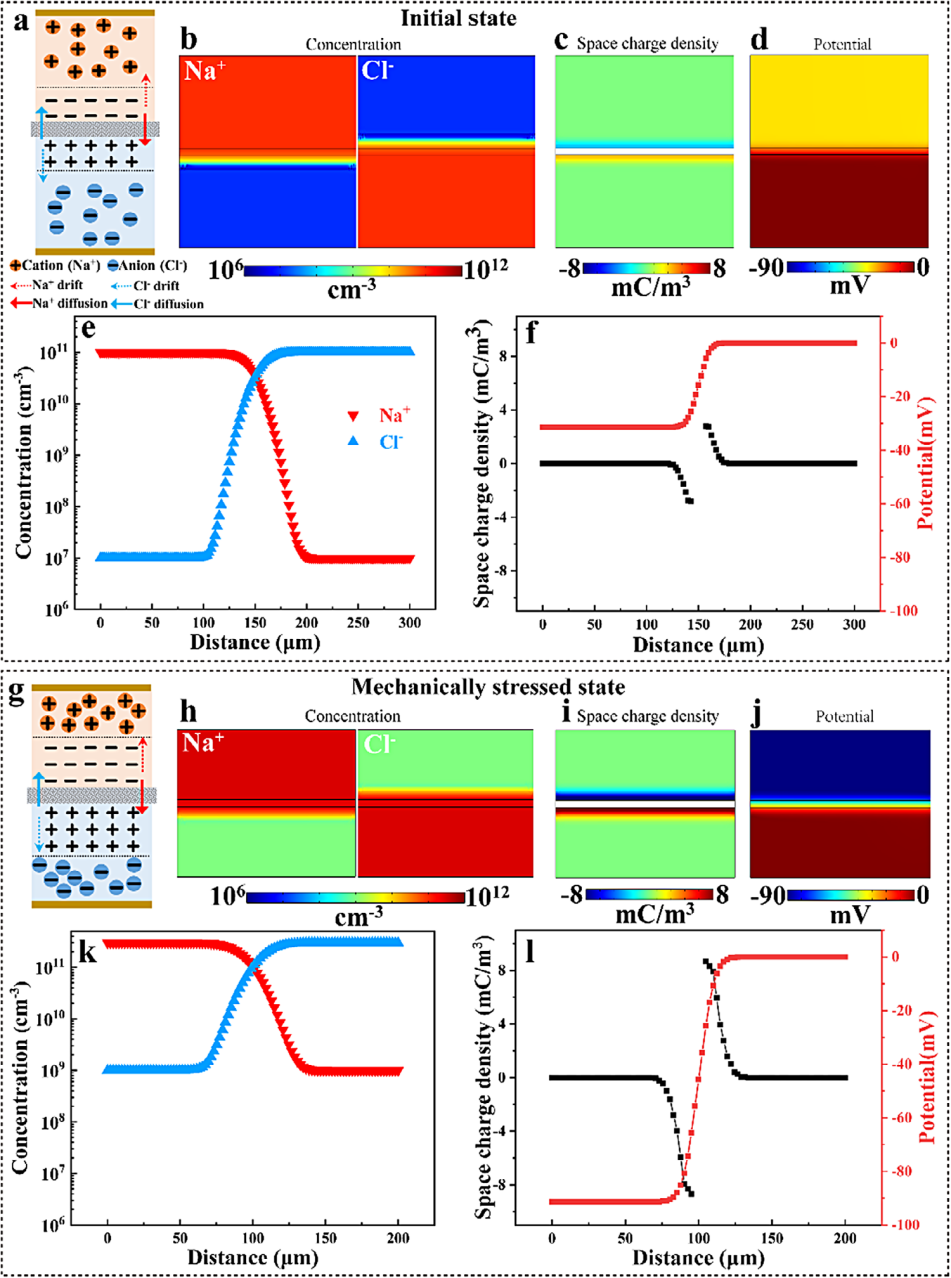
(a) Under the initial state or the diminishing stress and the mechanically released state, illustration showing the electrical behavior and response of the ionic diode. (b,e) Concentration profiles of Na^+^ and Cl^−^, (c,d,f) Space charge density, and potential gradient within the ionic diode simulated in the initial and released state. (g) Under external mechanical stress, the principle of electrical status of ionic diode with circuital charge flow. h,k) Concentration profiles of Na^+^and Cl^−^, (i,j,l) Space charge density, and potential gradient within the ionic diode simulated in the stressed state.

The working principle and simulation results of the A/B model, in which PSSNa and PDACl are positioned on opposite sides, are illustrated in Figure 2. In the initial state (Figure 2a,b), Na^+^ and Cl^−^ ions diffuse toward opposite sides, leaving uncompensated PSS^−^ and PDA^+^ backbones in their respective regions. Over time, a reverse internal electric field develops, driving the ions in the opposite direction and preventing further diffusion. Once the diffusion and drift motions reach equilibrium, a stable depletion layer is formed, establishing electrostatic balance (Figure 2c). The formation of the potential barrier is corroborated by finite element method (FEM) simulations using COMSOL (Figure 2d–f), which reveal a charge distribution within the depletion region analogous to that of a semiconductor p–n junction (Note S2). The quantitative ion concentration profiles derived from FEM simulations further confirm this mechanism (Figure 2e).

When an external mechanical force is applied (Figure 2g), the ion concentrations on both sides increase, thereby enhancing both diffusion and drift processes (Figure 2h,k). Consequently, an intensified internal electric field is generated in response to the applied stress (Figure S6). The corresponding increase in interfacial space charge density (Figure 2i,l) leads to a variation in the electrostatic potential (Figure 2j,l) [34]. Upon release of the external stress, the ions return to their initial equilibrium positions, allowing the ion concentration, space charge density, and potential distributions on both sides to recover to their original states.

**FIGURE 3:**
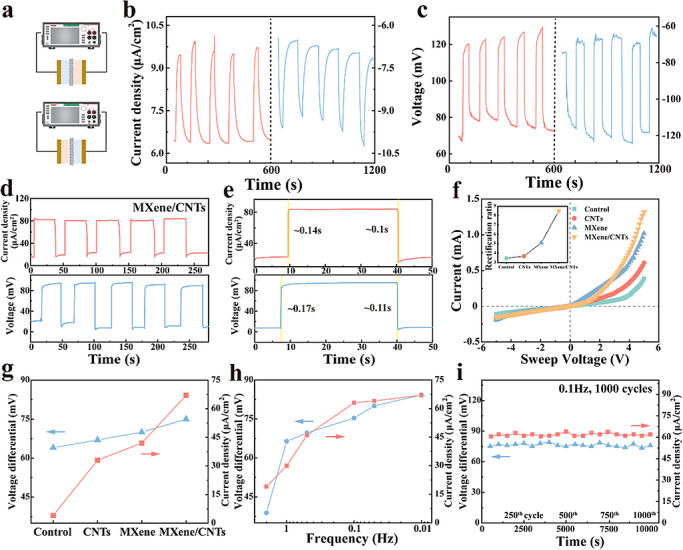
(a–c) Schematic illustration of energy‐harvesting measurement with a forward connection for current and voltage signals. (d) Current and voltage signal diagrams of components containing MXene/CNTs and (e) its local signal amplification. (f) Rectification performance of different components under 0.1 V s^−1^ scan rate. (Inset: rectification ratio of each component). (g) Voltage and current output of different components. (h) Voltage and current output at various input mechanical frequencies. (i) Durability test of the ionic‐diode (1000 cycles, 0.1 Hz).

Figure 3a–c present the voltage and current outputs of the hydrogel device under forward and reverse connections, respectively, showing identical magnitudes but opposite polarities. This symmetrical response confirms that the electrical signals originate from ion transport within the hydrogel pair, rather than from other effects such as triboelectrification. The output performance is intrinsically linked to the concentration of the polyelectrolytes (PSSNa and PDACl). These polymers provide the necessary mobile ions (Na^+^ and Cl^−^) and fixed charges to establish the ionic diode effect at the heterojunction interface. Variations in their content would alter the ion conductivity and the interfacial charge distribution, thereby modulating the output signal magnitude. When both sides consist of identical hydrogel components, only negligible potential fluctuations are observed under compression (Figure S7), further validating that the output arises from ionic migration. Incorporation of conductive 1D CNTs and 2D MXene fillers enhances internal ionic and electronic transport, yielding an optimized potential difference of approximately 75 mV and a short‐circuit current density of ∼67 µA cm^−^^2^ (Figure 3d,g). The single‐peak response of the hydrogel device exhibits a notably long duration (tens of seconds), much greater than that of conventional piezoelectric or triboelectric sensors (Figure 3e), indicating superior compatibility with low‐frequency human motion stimuli. To elucidate the underlying mechanism of this enhanced performance, the ionic conductivity of the hydrogel electrolytes was characterized using electrochemical impedance spectroscopy (EIS). As shown in Figure S8, the CNT/MXene hybrid hydrogel exhibits a significantly higher ionic conductivity of 40 mS cm^−^^1^. This substantial increase is attributed to the synergistic effect of the 1D CNTs and 2D MXene, which construct efficient ion transport pathways and prevent nanosheet restacking. This high ionic conductivity directly facilitates the rapid ion migration, thereby contributing to the superior current output observed in Figure 3 g.

The superior performance is attributed to the synergistic effect of the 1D/2D hybrid network. As illustrated in Figure 3f, MXene serves as the primary conductive component, while the incorporation of CNTs prevents MXene restacking and bridges isolated conductive domains. This architecture achieves a high rectification ratio of ∼8.8 compared to single‐component controls (Figure S21). This distinct rectification behavior serves as strong experimental evidence for the directional migration and accumulation of ions at the heterojunction interface. According to the ionic diode theory [47, 48], the macroscopic rectification effect originates from the formation of ion depletion and enrichment zones (concentration polarization) at the potential barrier. Therefore, the observed electrical signature validates the ion migration mechanism proposed in our FEM simulations, confirming the actual existence of the potential barrier and ion transport dynamics.

Based on the signal characteristics, each output peak can be divided into four stages associated with ion diffusion, charge accumulation, and potential variation (Figure S9). The first stage represents the equilibrium state following device assembly, during which ion motion is stabilized. In the second stage, the signal sharply increases as mechanical pressure induces rapid hydrogel deformation and accelerated ion diffusion between the layers. The third stage, characterized by a prolonged plateau, reflects a dynamic equilibrium state where the concentration‐driven diffusion flux is counterbalanced by the mechanical stimulation‐driven drift flux. Critically, the duration of this plateau is dictated not by ionic diffusion coefficients, but by the viscoelastic relaxation kinetics of the polymer network. This interpretation is supported by the stark contrast between our device and the unfilled PSS/PDAC ionic diode reported by Cayre et al., which exhibited a monotonic transient decay without any extended plateau [49]. In our system, the homogeneously dispersed 1D CNTs and 2D MXene nanosheets serve as additional physical crosslinking sites that significantly increase the crosslinking density of the hydrogel network [50, 51], thereby prolonging the characteristic relaxation time. Direct experimental evidence confirms that such nanofillers enhance both the storage modulus (G′) and loss modulus (G″) of hydrogels [52]. Consequently, the slow rearrangement of polymer chains—described by a poroelastic time constant that depends explicitly on the network's shear modulus and permeability—acts as the rate‐limiting kinetic barrier, physically constraining the reverse diffusion of ions. The final stage corresponds to recovery after stress release, as the hydrogel volume and internal ionic distribution return to equilibrium.

Notably, the hydrogel device exhibits a pronounced electromechanical response even at extremely low frequencies (0.01–0.1 Hz) (Figure 3 h; Figure S10), effectively overcoming the limitations of piezoelectric and triboelectric counterparts in the human‐motion frequency regime. The achieved current density of ∼67 µA cm^−^^2^ ranks among the highest reported for ionic‐diode‐based systems operating below 1 Hz. The dependence of voltage and current density on load resistance (Figure S11) allows the calculation of a maximum power density of ∼2.15 µW cm^−^^2^. The device's response to varying mechanical stresses (0–50 kPa) is shown in Figure S12: the output increases proportionally with applied pressure until plastic deformation occurs, after which a gradual decline is observed. To validate the long‐term stability required for practical applications, we extended the durability test to 1,000 loading‐unloading cycles (0.1 Hz, 50 kPa). As shown in Figure 3i, the device exhibits excellent robustness with negligible signal decay throughout the entire test duration. This remarkable durability stems from the ionic diode's intrinsic mechanism. Unlike electronic sensors prone to fatigue, our device relies on reversible ion diffusion rather than physical contact.

### 2.2 AB/0 Type Stack

The second configuration, designated as the AB/0 model, consists of PDACl and PSSNa hydrogels on one side and pure agarose on the other. As shown in Figure S13, the ionic radii of Na^+^ and Cl^−^ are 0.102 and 0.181 nm, respectively. Due to polymer chain entanglement and restricted ion convection within the hydrogel network, the migration of Na^+^ and Cl^−^ ions is hindered, and their size difference results in distinct migration velocities, thereby generating a potential difference [53]. The ionic steric hindrance model (Figure S13) effectively describes the movement of differently sized anions and cations within the agarose hydrogel. When the hydrogel undergoes initial deformation, the electrolyte is expelled from the compressed region, while the ions migrate at different rates, leading to charge separation (Figure S14b). Over time, this separation diminishes as reverse diffusion and electrophoretic effects occur (Figure S14c). The correlation between the potential difference (ΔV) and the applied external stress (σ) follows the relationship described by the following equation [23]:

(1) $$j=\sigma E+eN\left[v_{+}-v_{-}\right]=\sigma E+eNv\left[\frac{D_{+}}{D_{o+}}-\frac{D_{-}}{D_{o-}}\right]$$

where e is the electronic charge, N is the number of mobile cations per unit volume (assumed to be equal to the number of mobile anions), σ is the ionic conductivity, E is the electric field, v_+_ and v_‐_ represent the speed of movement of cations and anions, respectively. D_o+_ and D_o‐_ are the diffusion coefficients induced by the solution, and D_+_ and D_‐_ are the diffusion coefficients of the cations and anions in the hydrogel, respectively.

In this model, the upper layer comprises two ionic polymers, while the lower layer consists of pure agarose hydrogel. When the two types of ions (Na^+^ and Cl^−^) diffuse toward the same direction, the disparity in their diffusion coefficients gives rise to a voltage potential. A larger difference in diffusion coefficients results in a higher generated potential. Under an external pressure of 50 kPa, the device exhibits a potential difference of approximately 10 mV and a current density of 0.5 µA cm^−^^2^ (Figure S15a,b). The variations in current and voltage outputs under different mechanical loads, controlled by applied weights, are presented in Figure S15c. The results demonstrate that increasing external pressure enhances both the potential difference and current density, thereby establishing the fundamental basis for pressure‐sensing functionality.

### 2.3 A_1_/A_2_ Type Stack

A single‐ion migration model (A_1_/A_2_ model) was constructed by introducing different concentrations of PSSNa on both sides, thereby generating an electric potential through the diffusion of Na^+^ ions (Figure S16a). In this configuration, ion migration is driven not only by the Na^+^ concentration gradient but also by the internal electric field established between the fixed anionic polymer host (PSS^−^) and the mobile counterion (Na^+^). The migration behavior of Na^+^ ions within the hydrogel can be quantitatively described by the Nernst–Planck equation, as expressed below:

(2) $$j_{i}\left(x\right)=j_{i-diffusion}+j_{i-drift}=-D_{i}\nabla C_{i}\left(x\right)-z_{i}u_{i}FC_{i}\left(x\right)\nabla \varphi \left(x\right)$$

where j_i_(x) is the flow rate of substance i at x, D_i_ is the diffusion constant, ∂C_i_(x) is the concentration gradient at x, ∂φ(x) is the potential gradient at x, F is the Faraday constant, z_i_ is the valence state of the substance, u_i_ is the mobility related to the diffusion coefficient according to Nernst‐Einstein relationship u_i_ = D_i_/RT, R is the general gas constant, and T is the temperature, respectively.

When the two motion contributions are the same, the ion movements are in a steady state (j_i_(x) = 0). That is:

(3) $$\nabla C_{i}\;\left(x\right)=-\frac{Z_{i}F}{RT}C_{i}\left(x\right)\nabla \varphi \left(x\right)\;$$

(4) $$\Delta \;\varphi \;\left(x\right)=-\frac{Z_{i}F}{RT}\Delta lnC_{i}\left(x\right)\;$$

where the device is subjected to compressive stress, the concentration in the high concentration region increases more, thus Na^+^ ions would diffuse to the low concentration region. Therefore, the concentration difference of the two regions could response to compressive stress, which lay the foundation of mechanical energy harvesting and stress sensing. Similarly, PDACl could also be utilized for constructing the A_1_/A_2_ model, which taking advantage of the directional immigration of Cl^−^ ions under external stress.

As discussed above, the operating principle of this model is governed by ionic diffusion. Figure S17a,b present the electrical characteristics of the device fabricated with PSSNa as the active material under forward and reverse connections. In the initial state, a potential difference of approximately 13.2 mV is observed. Upon application of external mechanical stress, the potential decreases to ∼11.2 mV, accompanied by a current variation of about 50 nA cm^−^^2^ (Figure S17c). The potential difference varies with the magnitude of external stress, reaching a maximum change of ∼2 mV at an applied pressure of 50 kPa (Figure S17d). Excessive stress beyond this threshold induces irreversible hydrogel deformation, leading to significant performance degradation. Accordingly, the maximum applied pressure in this study was set to 50 kPa, consistent with the Young's modulus of PSSNa and PDACl.

Furthermore, the concentration gradient between the two hydrogel layers also influences the potential difference. As shown in Figure S17e, the potential increases with the concentration difference across the two sides, attaining optimal performance at a concentration disparity of 5 wt%. A similar trend is observed in the PDACl‐based device, as illustrated in Figure S17f.

### 2.4 A/A Type Stack

As discussed earlier, compressive deformation induces uniform strain on both sides of the device; therefore, chemical concentration asymmetry between the two layers is essential to generate directional ion migration and corresponding electrical response to external compression. In contrast, bending deformation results in tensile and compressive strain on opposite sides. When bending occurs, the ion concentration increases in the compressed region and decreases in the tensile region, leading to ion diffusion from the compression side toward the tensile side.

The schematic representation of the A/A model is illustrated in Figure S18a, where both sides consist of identical hydrogels with equal initial ion concentrations. Upon downward bending, the lower hydrogel layer experiences compression, causing an increase in ion concentration, while the upper layer undergoes tension, resulting in a decrease in concentration. Consequently, ions migrate from the lower (compressed) layer to the upper (tensile) layer, driven by the chemical potential difference between the two regions. The evolution of potential throughout a complete mechanical cycle is depicted in Figure S18b, showing that the potential difference arises from the distinct mechanochemical responses of the two layers. Corresponding FEM simulations (Figure S19) further illustrate the distributions of potential, space charge density, and ion concentration, corroborating the experimental observations.

As aforementioned, bending represents an asymmetric deformation process that generates a driving force for ion migration even within a structurally symmetrical device. By controlling the bending angle, distinct output signals corresponding to different deformation states can be obtained. As shown in Figure S20a,b, the potential difference increases progressively with increasing bending angle. Leveraging this property, the A/A model serves as an ideal candidate for bending‐mode recognition. As demonstrated in Figure S7, this homogenous structure exhibits minimal response to uniform compressive stress. This distinct insensitivity to pressure allows the A/A model to function as a “dedicated” bending sensor, thereby physically eliminating signal interference from normal pressure in multimodal sensing scenarios. The detailed fabrication process and structural configuration will be described later.

Figures S20c and d display the 3D output mappings of each module in the sensor array during upward and downward wrist bending, respectively. Notably, the modules located at the curved joint positions exhibit stronger electrical responses than those positioned elsewhere. The 3D potential distribution closely corresponds to the actual joint motion profile, confirming that the sensor array effectively identifies both the direction and angle of bending. Increasing the number of modules within the array further enhances the precision of motion recognition.

### 2.5 Rectifier Applications

**FIGURE 4:**
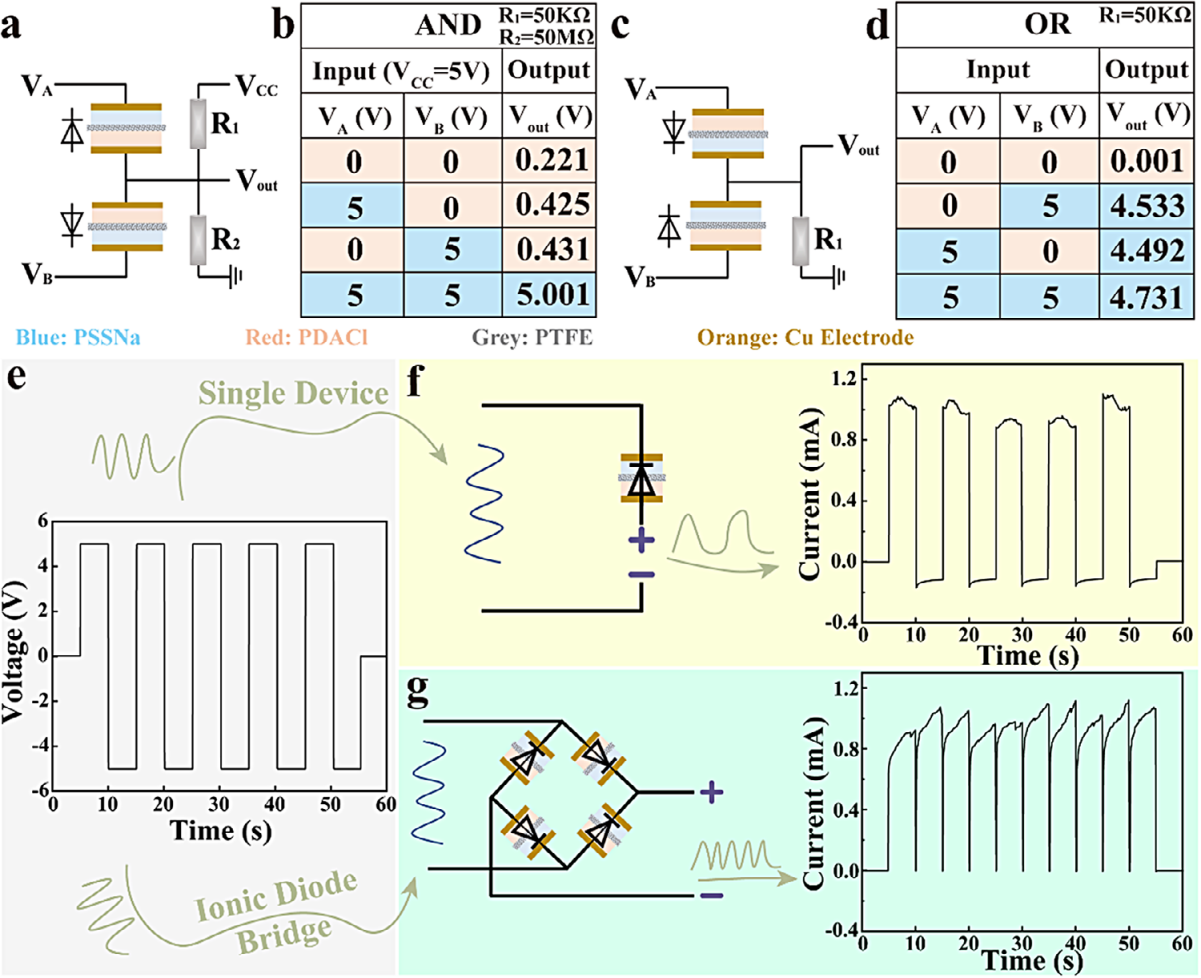
(a) Schematic of an “AND” gate fabricated by two hydrogel ionic diodes. (b) Truth table of the input and output signals of the “AND” gate. (c) Schematic of an “OR” gate fabricated by two hydrogel ionic diodes. (d) Truth table of the input and output signals of the “OR” logic gate. (e) AC square wave input signal. f) Schematics and output signal of the ionic rectifier integrating single hydrogel ionic diodes. g) the ionic Full‐bridge rectifier integrating four hydrogel ionic diodes.

Among the various models, the A/B configuration exhibits not only the highest electromechanical performance but also superior rectification characteristics, enabling its use as a diode component in “AND” and “OR” logic circuits. An alternating voltage ranging from −5 V to +5 V was applied to the hydrogel device at a sweep rate of 0.1 V s^−^^1^. As illustrated in Figure 4a, for the “AND” logic circuit, the fixed input terminal (Vcc = 5 V) is connected to the ground through two load resistors (R_1_ = 50 kΩ, R_2_ = 50 MΩ). The two input terminals, Va and Vb, correspond to the n‐junction sides of the two diodes, while the p‐junction terminals are connected to the midpoint between the resistors and the output terminal. In the schematic, the pink and blue blocks denote the cationic (PDACl) and anionic (PSSNa) hydrogel diodes, respectively. The orange and grey layers represent the Cu electrodes and PTFE spacer. The output potential is determined by controlling the voltages applied at Va and Vb (Figure 4b). When both inputs are 0 V or only one terminal is 5 V, the output voltage remains below 0.5 V, representing a “low” logic state. When both input voltages are 5 V, the output rises to 5.001 V, corresponding to a “high” logic state.

In the “OR” logic circuit (Figure 4c), the two input terminals are connected to the p‐sides of the hydrogel diode stack, whereas the n‐sides serve as output terminals and are grounded through a resistor (R_1_ = 50 kΩ). When both inputs are 0 V, the output voltage is approximately 0.001 V, indicating a “low” state. In contrast, when one or both inputs are 5 V, the output exceeds 4.5 V, signifying a “high” logic state (Figure 4d).

Figure 4e illustrates the AC square wave input signal applied to the devices. Figure 4f shows the schematic of the single‐device rectifier and its corresponding output current response. As shown in the current plot of Figure 4f, the device exhibits clear rectification behavior, where the forward current is about 5 times greater than the reverse leakage current, confirming its directional conductivity. Building upon this, Figure 4g demonstrates the full‐bridge rectifier configuration using four such devices, which successfully converts the AC input into a pulsating DC output.

Furthermore, the incorporation of conductive fillers significantly enhances the rectification performance. The rectification ratio of the MXene/CNT composite device reaches as high as 8.8. In contrast, the control sample fabricated without conductive fillers (Figure S21) exhibits no current reversal under alternating bias, confirming the absence of rectifying functionality in the pure hydrogel component.

### 2.6 Sensing Application

Owing to its high power density under extremely low‐frequency bending and compressive stimuli, the ionic‐diode device represents a promising candidate for self‐powered and high‐fidelity pressure and tactile sensing applications. Figure S22 illustrates the charging process of the ionic‐diode device under continuous mechanical pressing, during which the generated electrical energy is stored in a capacitor to power miniature wearable electronics.

**FIGURE 5:**
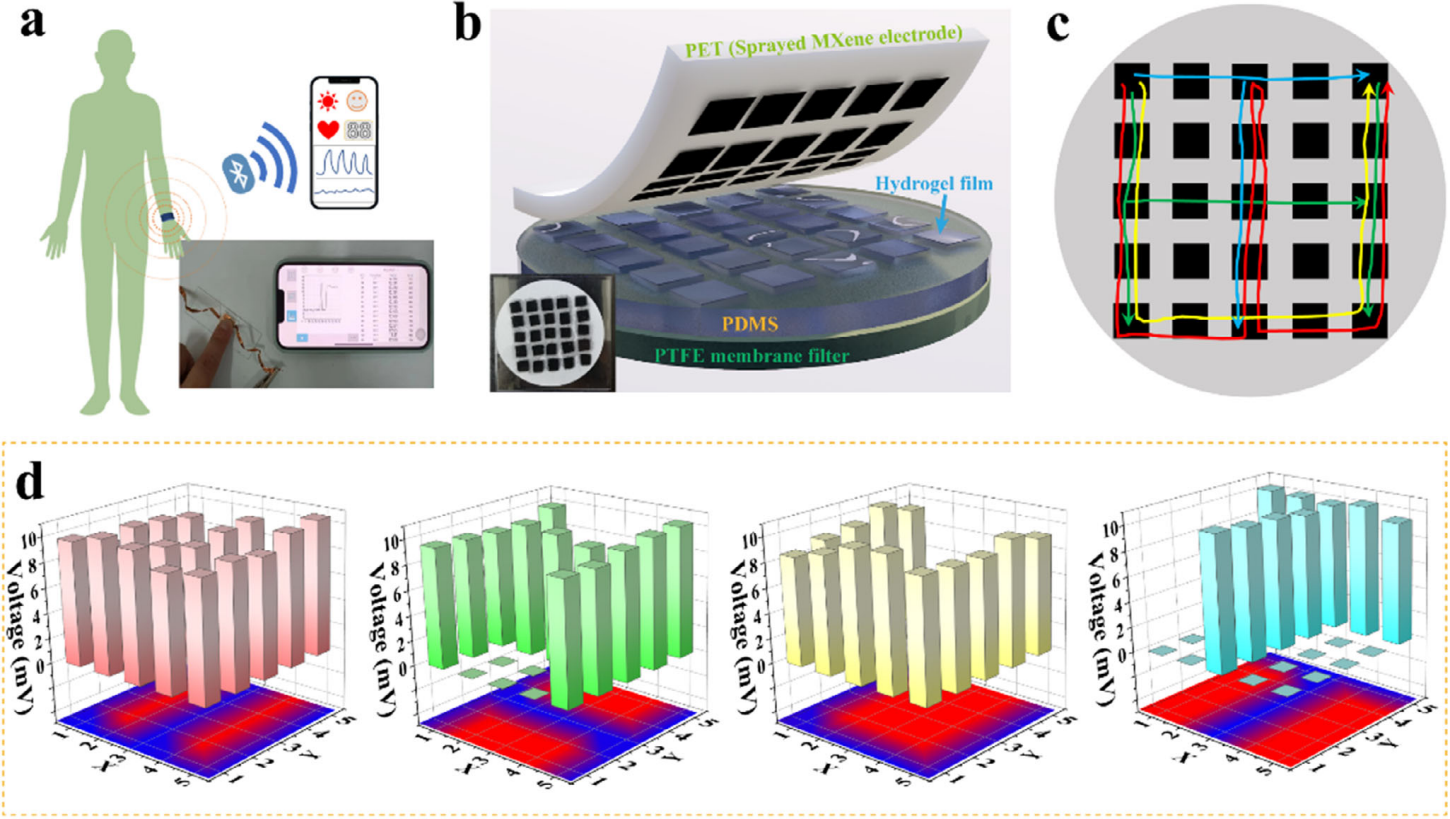
(a) Schematic and physical diagram of wireless sensing of test signals to mobile phones. (b) Schematic of the hydrogel ionic diodes sensor array (Inset: Physical diagram of hydrogel arrangement in array). (c) The motion trajectory of the stimulation on the sensor array and (d) the corresponding electrical performance output.

The collected sensing signals can be wirelessly transmitted to mobile devices via a Bluetooth interface (Figure 5a), enabling convenient real‐time monitoring for practical applications. The sensing resolution and spatial accuracy can be further enhanced by assembling multiple devices into a sensor array, as shown in Figure 5b. The array is fabricated by embedding hydrogels into 5 × 5 square cavities within a PDMS matrix. MXene serves a dual function—acting as a conductive filler to form efficient charge‐transport pathways and simultaneously as an electrode material. As shown in Figure S23a,b, MXene solution is spray‐coated onto PET substrates through a patterned mask to form the top electrodes, exhibiting a high electrical conductivity exceeding 1 × 10^4^ S cm^−^^1^. To address the environmental instability of hydrogels, a PDMS encapsulation layer is applied. As summarized in Figure S24, unencapsulated devices suffer from severe dehydration, leading to a significant drop in output voltage from ∼75 to ∼10 mV and current to 0.5 µA cm^−^^2^ within 48 h. Detailed comparisons of physical properties (Figure S24b,c) reveal that this severe water loss causes marked reductions in area, thickness, weight, and ionic mobility. In contrast, the PDMS layer effectively suppresses dehydration, thereby preserving the device's functional integrity and long‐term stability.

The fully integrated device is assembled on a filter membrane substrate, allowing rapid and stable interconnection among individual modules to ensure consistent performance. Additionally, the PDMS encapsulant firmly fixes the hydrogel in place, preventing slippage and minimizing crosstalk between adjacent sensing units. Figure 5c presents four sliding trajectories on the sensor array forming the letters “WHUT,” while the corresponding electrical responses from each module (Figure 5d) exhibit clearly distinguishable potential variations, confirming the array's excellent spatial sensing capability and signal fidelity.

As discussed above, the A/B, A_1_/A_2_, and AB/0 models demonstrate distinct advantages in identifying compressive deformation, whereas the A/A configuration is particularly suitable for detecting bending stimuli. Multimodal deformation can thus be recognized by integrating multiple types of devices into a unified sensing array. By calculating the potential difference ( ΔV ) between paired modules, the system can effectively decouple the deformation modes. This orthogonal design—combining pressure‐sensitive A/B models with bending‐sensitive A/A models—allows the array to distinguish multimodal stimuli based on their distinct spatial “fingerprints”, even though the raw output of each unit is a voltage signal.

**FIGURE 6:**
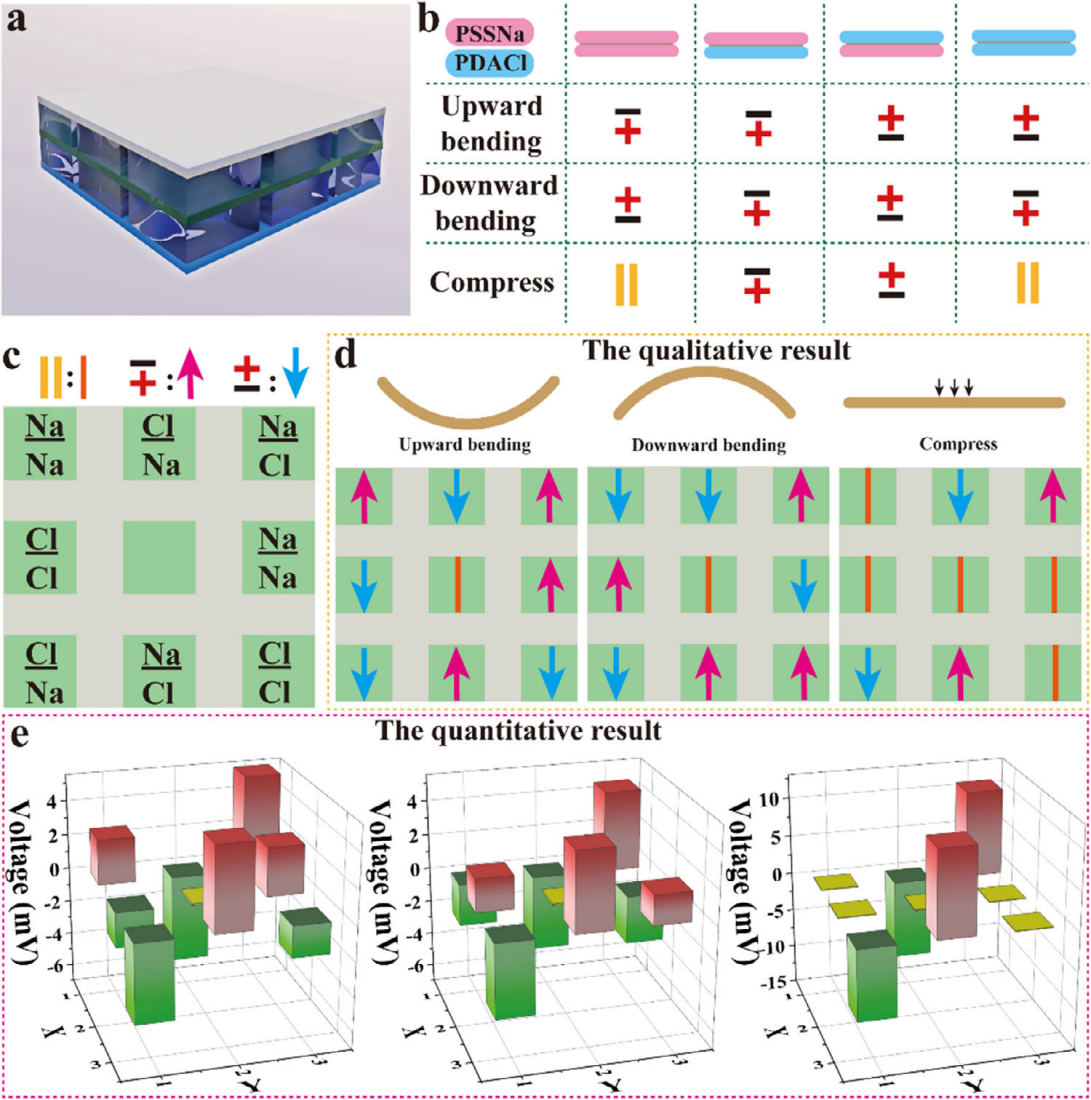
(a) Schematic of the hydrogel ionic diodes permutation combination sensor array sensor array. (b) Potential distribution of different models under different external stimulation. (c) Distribution of different models in permutation sensor array. (d) Qualitative and (e) Quantitative results of the permutation sensor array under different external stimulation.

As illustrated in Figure 6a, a 3 × 3 micromodule array was assembled into an integrated sensing unit. The structural composition of each module is detailed in Figure 6c, comprising four bending‐recognition modules (PSSNa/PSSNa and PDACl/PDACl) and four compression‐recognition modules (PSSNa/PDACl), with two of the latter positioned in opposite orientations. The collective performance of the sensor array—composed of these distinct module types characterized in Figure 6b—was qualitatively evaluated under various deformation conditions (Figure 6d). The arrows in the output maps indicate the direction of potential generation, and the combination of signal vectors enables determination of the overall deformation state. By analyzing the potential amplitude from each micromodule, the extent of deformation can be quantitatively assessed (Figure 6e). The corresponding bar charts intuitively represent both the magnitude and polarity of the signals, thereby enabling simultaneous identification of deformation direction and intensity.

**FIGURE 7:**
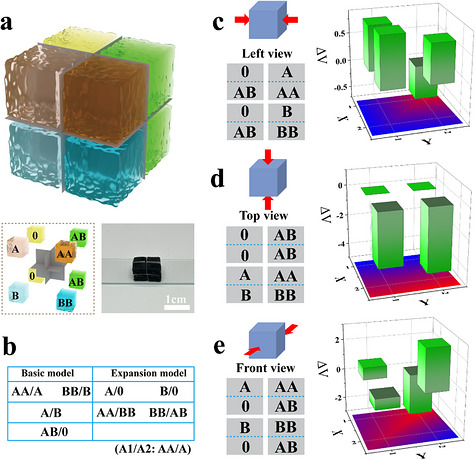
(a) Schematic diagram and digital picture of the Rubik's cube pressure sensor. (b) The used four models. (c–e) The left, top and front view and corresponding potential bar chart.

However, while the planar array validates the basic principle, it is limited to 2D surface sensing. The capability to discern stress direction in 3D space remains largely unachieved in previously reported sensors. To address this limitation and fully exploit our structural diversity, we designed a Rubik's cube–inspired sensor array (Figure 7a), in which all four model types are rationally integrated into a 3D architecture. Unlike conventional planar arrays that rely on spatial separation, this 3D architecture utilizes structural multiplexing. As shown in Figure 7c–e, when stimulated along different orthogonal directions, the specific combination of units activated generates a unique potential mapping. The A/B units provide high‐contrast rectification for directional identification. The AA/A (A_1_/A_2_) and AB/0 units contribute to distinguishing bending versus compression. This design demonstrates that by rationally permuting the four basic structural units, we can create a sensor system capable of unambiguously identifying the directionality and mode of mechanical stimuli in 3D space—a capability rarely achieved in previous self‐powered tactile sensors.

## 3 Conclusion

Inspired by ion transport mechanisms in the human tactile system, we developed self‐powered ionic‐diode pressure sensors composed of two types of hydrogels containing mobile anions and cations. Finite element method (FEM) simulations confirmed the formation of a depletion layer at the hydrogel interface, where the built‐in potential dynamically varies under external mechanical stimulation. Incorporation of conductive 1D CNTs and 2D MXene nanosheets significantly enhanced ion mobility and consequently improved the electromechanical coupling performance. Among the four designed models, the A/B configuration exhibited the highest mechanical energy‐harvesting efficiency at extremely low frequencies, along with excellent rectification and sensing characteristics. A maximum power density of ∼2.15 µW cm^−^^2^ was achieved at frequencies below 0.1 Hz. Furthermore, these four models were integrated into sensing arrays capable of distinguishing multiple deformation modes—including compression, bending, and even stress direction—representing the first demonstration of such multifunctional capability. This work establishes an ion‐transport–based strategy for designing high‐performance, self‐powered tactile sensors. Moving beyond simple trajectory recognition, our “permutation sensor array” provides a high‐fidelity, multi‐dimensional perception capability (including pressure, bending, and directional stress) within a single device. This advancement offers a new paradigm for next‐generation wearable electronics and soft robotics, where comprehensive environmental feedback is crucial for intelligent decision‐making.

## 4 Experimental Section

### 4.1 Gel Preparation

7 wt% Sodium polystyrene sulfonate (PSSNa, 70 kDa, Sigma Aldrich) was dissolved in deionized water and magnetically stirred for 2 h, then 1D carboxylated carbon nanotubes (CNTs, Sigma Aldrich) and 2D MXene (Jilin 11 Technology Co.) were added and mixed for another 3 h. For the composite‐doped hydrogel, the loading was optimized at 2.5 wt% CNTs and 2.5 wt% MXene (relative to the hydrogel matrix). For the single‐doped control groups, the samples contained either 5 wt% CNTs or 5 wt% MXene to maintain a consistent total filler concentration. Agarose (Sigma Aldrich, BioReagent, formolecular biology, low electroendosmosis, A9539) with a mass fraction of 6 wt% was added to above solution via magnetic stirring and then heated to 130°C. The homogeneous solutions were transferred to glass plates and flattened. They were then naturally cooled to room temperature to form stable hydrogels via thermal‐induced physical gelation of agarose. Poly(diallyldimethylammoniumchloride) (PDACl, 400–500 kDa with 20 wt% solution in water) hydrogel was prepared via the same follow‐up method with the mass fraction of 5 wt%. Given the low loading of conductive fillers (CNTs/MXene), the resulting composite hydrogel possesses a high‐water content of approximately 87 wt.%–89 wt.%, which ensures sufficient ion mobility. The subsequent preparation method of pure component without ionic polymer is the same.

### 4.2 Device Assembly

The two gels were assembled together with a polytetrafluoroethylene (PTFE) permeable membrane (pore size of ∼1 µm, Sterlitech Co.) as the separator. Copper foils (thickness of ∼10 µm) were attached to each end of the two gels as the electrodes. The electrodes were connected via direct wire leads attached to the copper foils.

### 4.3 Characterizations

The morphology of MXene, CNTs and hydrogels were carried out using a field‐emission scanning electron microscope (FE‐SEM, JSM‐7610F, Japan) and transmission electron microscope (TEM, Talos F200S, Thermo Scientific, USA). The thickness and surface roughness of MXene were measured by atomic force microscope (AFM, Cypher ES, Asylum Research, USA) in a standard tapping mode. The crystal structure of powders was evaluated by X‐ray diffraction pattern (XRD, Rigaku Smartlab, Japan). XRD was also analyzed by Center for University‐Wide Research Facilities (CURF) at Jeonbuk National University. All electromechanical characterizations were performed under controlled laboratory conditions at a temperature of 20 ± 1°C and a relative humidity of 50% to ensure data consistency. The voltage and current signals generated by the device were measured by Keithley DMM7510, and the rectification performance is characterized by Keithley 6517b.

## Author Contributions

**Haoran Chen**: Conceptualization, Methodology, Formal analysis, Investigation, Data Curation, Writing – Original Draft, Visualization. **Hongjian Zhang**: Methodology, Formal analysis, Investigation, Data Curation, Writing – Original Draft, Visualization. **Zhonghui Shen**: Software, Methodology, Formal analysis, Visualization. **Taeuk Eom**: Methodology, Formal analysis, Visualization. **Hyunseung Kim**: Methodology, Validation, Data Curation, Visualization. **Delong He**: Formal analysis, Investigation. **Jinbo Bai**: Formal analysis, Investigation. **Geon‐Tae Hwang**: Resources, Formal analysis, Investigation. **Yong Zhang**: Conceptualization, Software, Validation, Investigation, Resources, Writing – Original Draft, Writing – Review & Editing, Supervision, Project administration, Funding acquisition. **Chang Kyu Jeong**: Conceptualization, Validation, Investigation, Resources, Writing – Original Draft, Writing – Review & Editing, Supervision, Project administration, Funding acquisition.

## Conflicts of Interest

The authors declare no competing financial interests.

## Supporting information




**Supporting File**: advs75848‐sup‐0001‐SuppMat.pdf.

## Data Availability

The data that support the findings of this study are available from the corresponding author upon reasonable request.
