# Supplementary material for: Hydrogel Stack‐Tailored Logics and High‐Fidelity Multimodal Sensors Promoted by Precisely Evaluated Ionic Migration
Source: Adv Sci (Weinh). 2026 Jun 4:e75848. Online ahead of print. doi: 10.1002/advs.75848 (PMC13335863; doi:10.1002/advs.75848)
Supplement: Supplementary file 1 — Supporting File: advs75848‐sup‐0001‐SuppMat.pdf. [file ADVS-9999-e75848-s001.pdf]

Supporting Information

Hydrogel Stack-Tailored Logics and High-Fidelity Multimodal Sensors Promoted by Precisely Evaluated Ionic Migration

Haoran Chen, Hongjian Zhang, Zhonghui Shen, Taeuk Eom, Hyunseung Kim, Delong He, Jinbo Bai, Geon-Tae Hwang, Yong Zhang, Chang Kyu Jeong**

H. Chen, H. Zhang, Z. Shen, Y. Zhang
State Key Laboratory of Advanced Glass Materials, School of Materials Science and Engineering, Wuhan University of Technology, Wuhan 430070, China

H. Chen, H. Zhang, Z. Shen, Y. Zhang
Center for Smart Materials and Device Integration, Wuhan University of Technology, Wuhan 430070, China

T. Eom, C. K. Jeong
Department of JBNU-KIST Industry-Academia Convergence Research, Jeonbuk National University, Jeonju, Jeonbuk 54896, Republic of Korea

T. Eom, H. Kim, C. K. Jeong
Division of Advanced Materials Engineering, Jeonbuk National University, Jeonju, Jeonbuk 54896, Republic of Korea

H. Kim, C. K. Jeong
Department of Energy Storage/Conversion Engineering of Graduate School (BK21 FOUR), Jeonju, Jeonbuk 54896, Republic of Korea

D. He, J. Bai
LMPS–Laboratoire de Mécanique Paris-Saclay, Université Paris-Saclay, CentraleSupélec, ENS Paris-Saclay, CNRS, Gif-sur-Yvette 91190, France

G.-T. Hwang
Department of Materials Science and Engineering, Pukyong National University, Busan 48513, Republic of Korea

*Corresponding author.
E-mail: zhangyong123@whut.edu.cn (Y. Zhang), ckyu@jbnu.ac.kr (C. K. Jeong)
Keywords: self-powered; sensor array; ionic diode; energy harvesting; logic circuit

1. Mechanical analysis of bending deformation

We can calculate the efficiency of the energy conversion by analyzing the bending forces. For the symmetric assembly structure, the bending deformation produces equal amount of tensile and compressive deformation on both sides of the hydrogel ($\varepsilon_{xx}=\pm h/R$), where R is the radius of curvature and h is the half-thickness of the thin-film device, and the force in each direction of one of the gels is:

$$\sigma_{xx} = \frac{Yh}{(1-v^2)R}, \quad \sigma_{yy} = 0, \quad \sigma_{ZZ} = \frac{vYh}{(1-v^2)R}$$

Where Y is Young's modulus, v is Poisson's ratio, the strain energy generated in the system can be coupled into hydrostatic and deviatoric stresses:

$$U_{strain} = U_{hydrostatic} + U_{deviatoric} = \frac{Yh^2}{2(1-v^2)R^2}$$

where,

$$U_{hydrostatic} = \frac{\sigma_{hydrostatic}^2}{2B}, \quad B = \frac{Y}{3(1-2v)}$$

B is the bulk modulus of the electrode. Assuming that only the hydrostatic stress component contributes to the energy production and all deviatoric components are consumed, the ideal efficiency is:

$$\eta = \frac{U_{electrical}}{U_{strain}} = \frac{U_{hydrostatic}}{U_{strain}} = \frac{(1-2v)(1+v)}{3(1-v)}$$

2. Simulation

The diffusion behavior of the ions and the local potential distribution can be obtained by solving the coupled transport equation as well as Poisson's equation, which can be described as,

$$\frac{\partial c_i}{\partial t} + \nabla \cdot (-D_i \nabla c_i - z_i \mu_i F c_i \nabla \varphi) = 0. \quad (1)$$

$$-\nabla^2 \varphi = \frac{(c_{PDAC^+} + c_{Na^+} - c_{PSS^-} - c_{CL^-}) N_A e}{\varepsilon_0 \varepsilon_r}. \quad (2)$$

where i, c_i, D_i, z_i, μ_i, F, φ, N_A, e, ε_0, ε_r represent the mobile species, the concentration, the diffusion coefficient, the charge number of the mobile species, the mobility, the Faraday constant, the local electric potential, the Avogadro constant, the element charge, the vacuum permittivity and the relative dielectric constant, respectively.

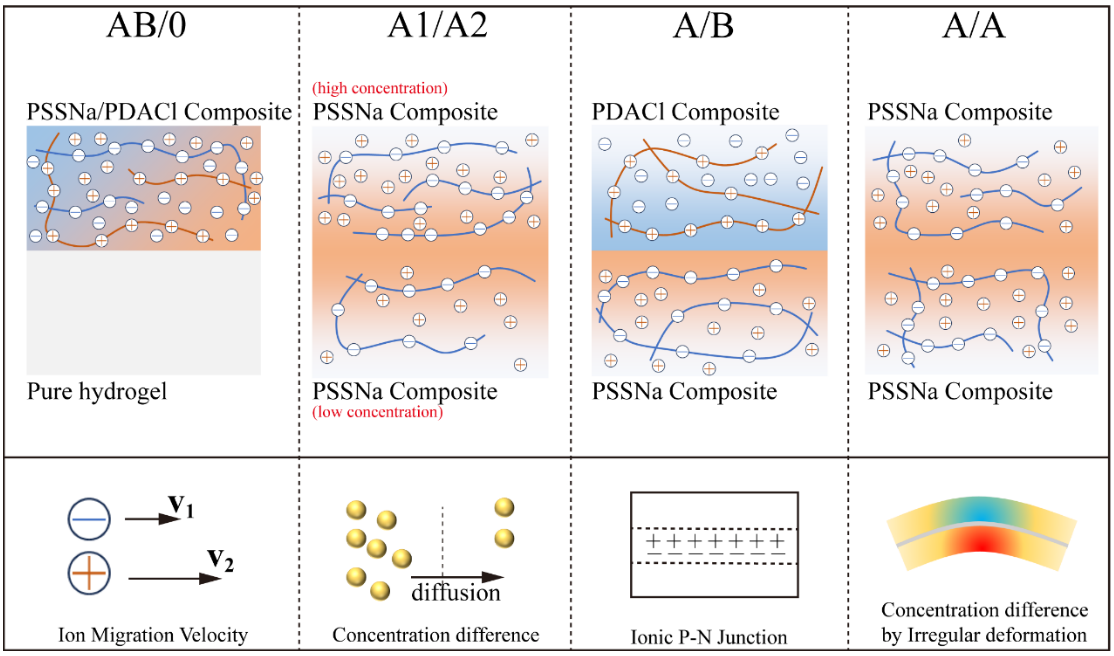

Figure S1. The configuration and mechanism of four types of ionic-diode junction based electromechanical device.

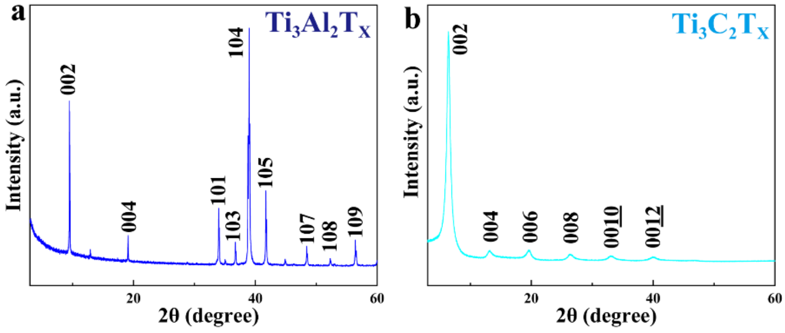

Figure S2. X-ray diffraction patterns of MAX phase a) before and b) after corrosion in HCl/LiF mixed solution.

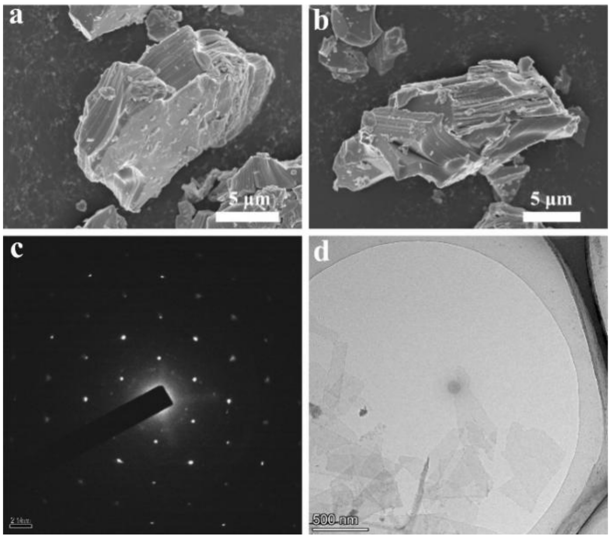

Figure S3. SEM images of dense Ti_3AlC_2 MAX powder (a) before and (b) after etching; c,d) TEM images of exfoliated MXene.

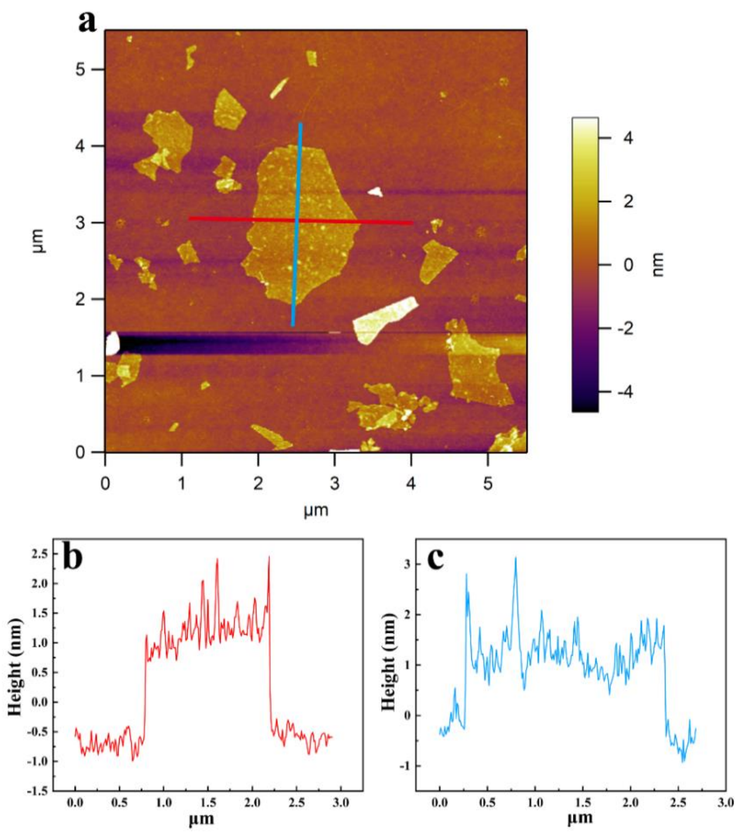

Figure S4. a) AFM spectra of exfoliated MXene. b) Transverse and (c) longitudinal thickness distribution of nanosheets

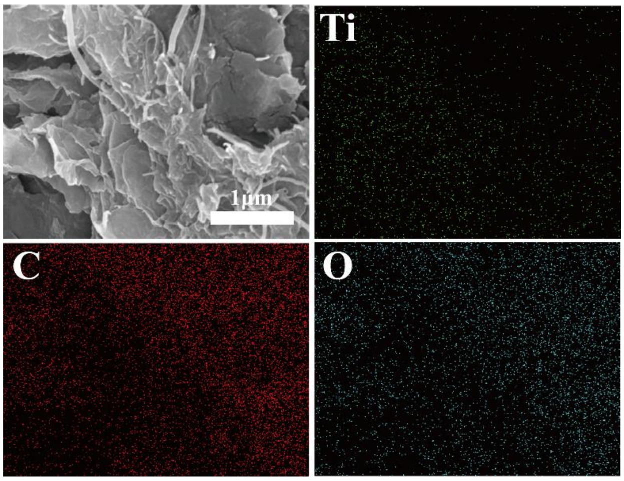

Figure S5. EDS mapping of Ti and C elements in hydrogels.

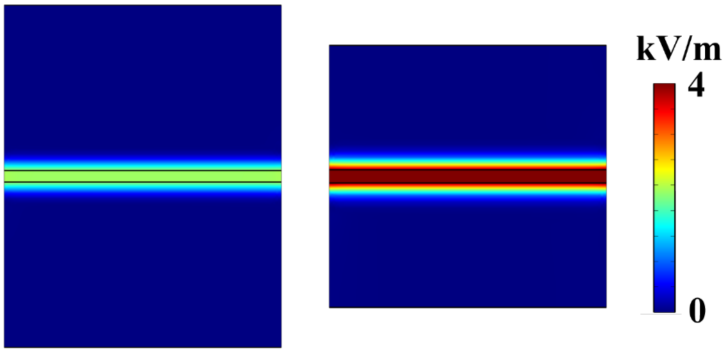

Figure S6. Electric field within the ionic-diode simulated in the initial & released state (left) and the stressed state (right).

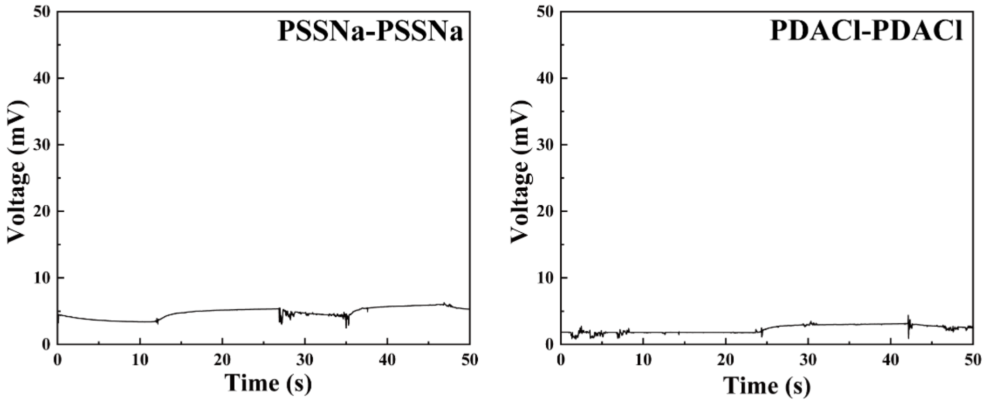

Figure S7. The potential voltage of ionic-diode device with the same hydrogel nanocomposites in both sides.

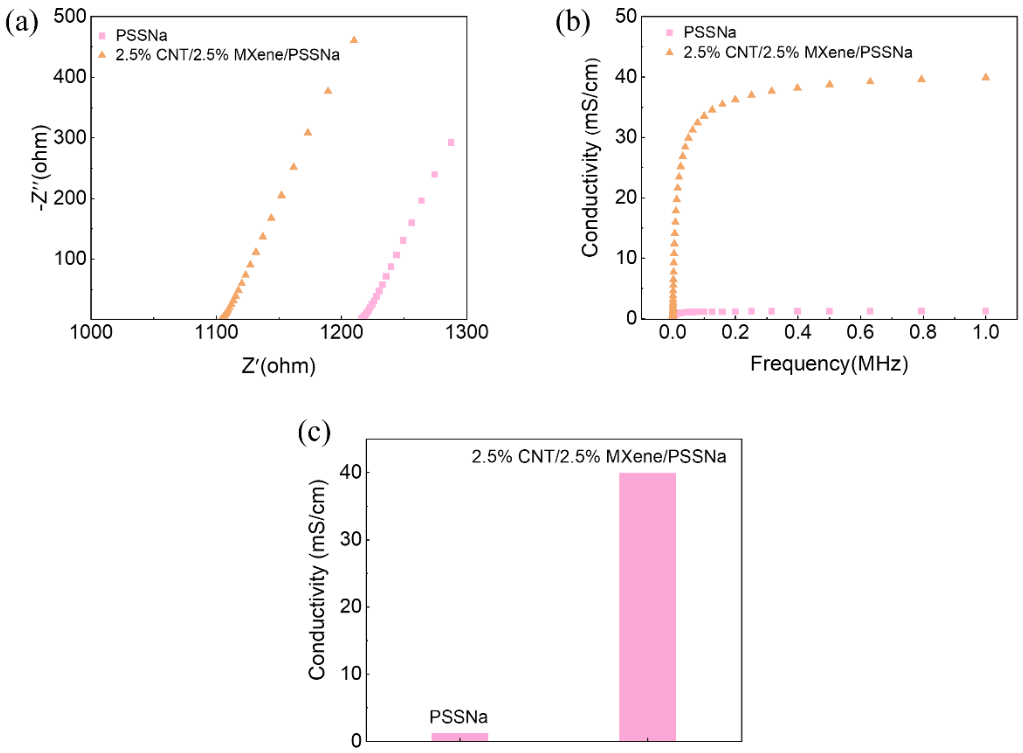

Figure S8. (a) Nyquist plots and (b) frequency-dependent conductivity spectra of PSSNa and 2.5% CNT/2.5% MXene/PSSNa hydrogels. (c) Calculated ionic conductivity values for the different hydrogel compositions.

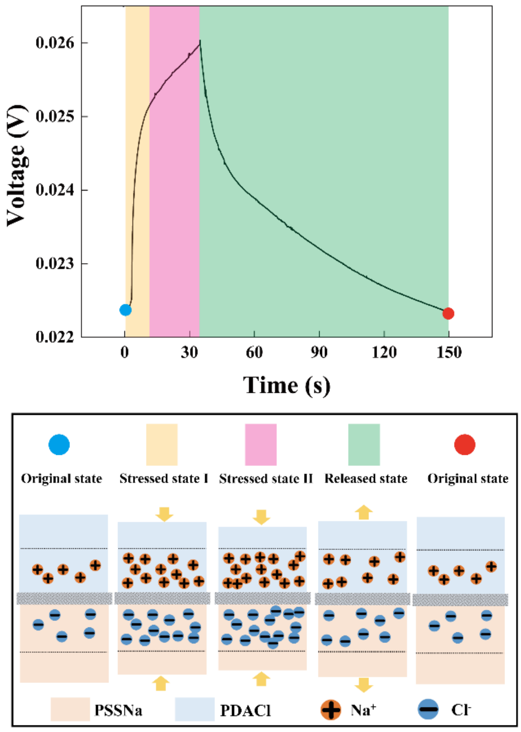

Figure S9. Detailed working mechanism of the ionic-diode divice.

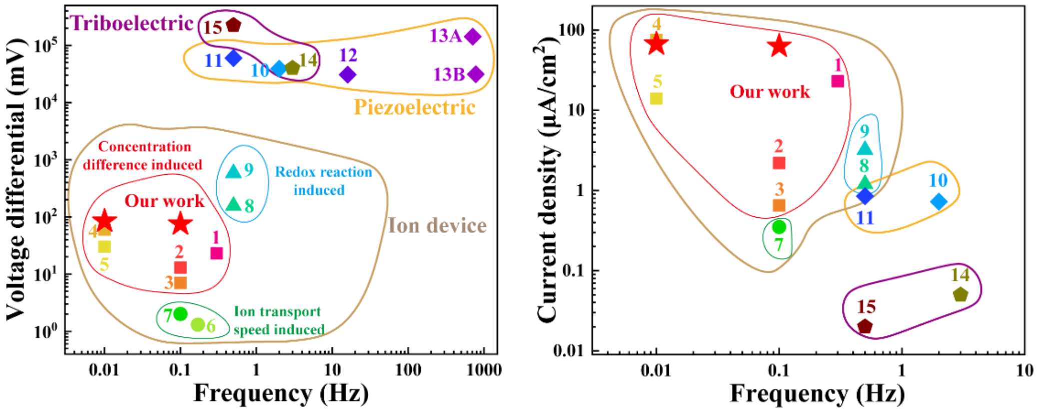

		Reference	Composition	Transmission media	Frequency (Hz)	Voltage (mV)	Current (μA/cm²)
Ion device	Concentration difference induced	1	Li-alloyed Si electrodes	Li^+	0.3	23	23
		2	2D Black Phosphorus Nanosheets	Na^+	0.1	13	2.2
		3	DF25/MWCNTs//Nafion/MWCNTs	BF_4^-, EMI^+	0.1	7	0.65
		4	PSSNa/CNT/AgNWs//PDACl/CNT/AgNWs	Na^+, Cl^-	0.01	60	75
		5	PSSNa/CNT//PDACl/CNT	Na^+, Cl^-	0.01	30	14
	Ion transport speed induced	6	H-RGO/CNTs/Ag, TPU/EMITFSI	$TFSI^-$, EMI^+	0.17	1.3	\
		7	Poly(AA-co-AAm)	$TFSI^-$, Li^+	0.1	2	0.35
	Redox reaction induced	8	PB/carbon//PVA/NaCl/Gly/H_2O//Ag/AgCl	$AFe^{II}Fe^{III}(CN)_6 + x\backslash A^+ + x\,e^- \leftrightarrow A_{1+x}Fe^{II}[Fe^{II}(CN)_6]_x[Fe^{III}(CN)_6]_{1-x}$, $Ag + Cl^- \leftrightarrow AgCl + e^-$	0.5	155	1.2
		9	rGO/GO/PVA nanofibers/MXene	$8H^+ + 8e^- = 4H_2$ $Ti_3C_2O_2 + 4H_2O - 8e^- = 3TiO_2 + 2C + 8H^+$	0.5	580	3.2
Piezoelectric		10	BTS-BCT	\	2	39×10³	0.725
		11	Sm-PMN-BT	\	0.5	60×10³	0.85
		12	PMNN-PZT	\	16	30.7×10³	\
		13A	PMN-PT	\	710	142×10³	\
		13B	PNN-PZT	\	780	31.2×10³	\
Triboelectric		14	CB-TPU	\	3	40×10³	0.05
		15	MXene/PVA	\	0.5	225×10³	0.02

Figure S10. Comparison between the previously reported ionic, piezoelectric, triboelectric device and this work in potential voltage and current density.

References:

1. S. Kim, S.J. Choi, K. Zhao, H. Yang, G. Gobbi, S. Zhang, J. Li, Electrochemically driven mechanical energy harvesting, Nat. Commun. 7 (2016) 10146.
2. N. Muralidharan, M. Li, R.E. Carter, N. Galioto, C.L. Pint, Ultralow Frequency Electrochemical-Mechanical Strain Energy Harvester Using 2D Black Phosphorus Nanosheets, ACS Energy Lett. 2 (2017) 1797–1803.
3. Y. Hou, Y. Zhou, L. Yang, Q. Li, Y. Zhang, L. Zhu, M.A. Hickner, Q.M. Zhang, Q. Wang, Flexible Ionic Diodes for Low-Frequency Mechanical Energy Harvesting, Adv. Energy Mater. 7 (2017) 1–6.
4. Y. Zhang, C.K. Jeong, J. Wang, X. Chen, K.H. Choi, L.Q. Chen, W. Chen, Q.M. Zhang, Q. Wang, Hydrogel Ionic Diodes toward Harvesting Ultralow-Frequency Mechanical Energy, Adv. Mater. 33 (2021) 1–9.
5. Y. Zhou, Y. Hou, Q. Li, L. Yang, Y. Cao, K.H. Choi, Q. Wang, Q.M. Zhang, Biocompatible and Flexible Hydrogel Diode-Based Mechanical Energy Harvesting, Adv. Mater. Technol. 2 (2017) 1–6.
6. J. Zhao, S. Han, Y. Yang, R. Fu, Y. Ming, C. Lu, H. Liu, H. Gu, W. Chen, Passive and Space-Discriminative Ionic Sensors Based on Durable Nanocomposite Electrodes toward Sign Language Recognition, ACS Nano. 11 (2017) 8590–8599.

7. Y. Dobashi, D. Yao, Y. Petel, T.N. Nguyen, M.S. Sarwar, Y. Thabet, C.L.W. Ng, E.S. Glitz, G.T.M. Nguyen, C. Plesse, F. Vidal, C.A. Michal, J.D.W. Madden, Piezoionic mechanoreceptors: Force-induced current generation in hydrogels, Science (80-.). 376 (2022) 502–507.
8. X. Wu, M. Ahmed, Y. Khan, M.E. Payne, J. Zhu, C. Lu, J.W. Evans, A.C. Arias, A potentiometric mechanotransduction mechanism for novel electronic skins, Sci. Adv. 6 (2020) 1–11.
9. D. Lei, Q. Zhang, N. Liu, T. Su, L. Wang, Z. Ren, Y. Gao, An Ion Channel-Induced Self-Powered Flexible Pressure Sensor Based on Potentiometric Transduction Mechanism, Adv. Funct. Mater. 32 (2022) 1–11.
10. H. Su, X. Wang, C. Li, Z. Wang, Y. Wu, J. Zhang, Y. Zhang, C. Zhao, J. Wu, H. Zheng, Enhanced energy harvesting ability of polydimethylsiloxane-BaTiO3-based flexible piezoelectric nanogenerator for tactile imitation application, Nano Energy. 83 (2021) 105809.
11. Y. Zhang, C.K. Jeong, J. Wang, H. Sun, F. Li, G. Zhang, L.Q. Chen, S. Zhang, W. Chen, Q. Wang, Flexible energy harvesting polymer composites based on biofibril-templated 3-dimensional interconnected piezoceramics, Nano Energy. 50 (2018) 35–42.
12. Z. Yu, J. Yang, J. Cao, L. Bian, Z. Li, X. Yuan, Z. Wang, Q. Li, S. Dong, A PMNN-PZT Piezoceramic Based Magneto-Mechano-Electric Coupled Energy Harvester, Adv. Funct. Mater. 32 (2022) 1–11.
13. X. Gao, J. Wu, Y. Yu, Z. Chu, H. Shi, S. Dong, Giant Piezoelectric Coefficients in Relaxor Piezoelectric Ceramic PNN-PZT for Vibration Energy Harvesting, Adv. Funct. Mater. 28 (2018) 1–8.
14. W. Zhang, Q. Liu, S. Chao, R. Liu, X. Cui, Y. Sun, H. Ouyang, Z. Li, Ultrathin Stretchable Triboelectric Nanogenerators Improved by Postcharging Electrode Material, ACS Appl. Mater. Interfaces. 13 (2021) 42966–42976.
15. X. Luo, L. Zhu, Y.C. Wang, J. Li, J. Nie, Z.L. Wang, A Flexible Multifunctional Triboelectric Nanogenerator Based on MXene/PVA Hydrogel, Adv. Funct. Mater. 31 (2021) 1–9.

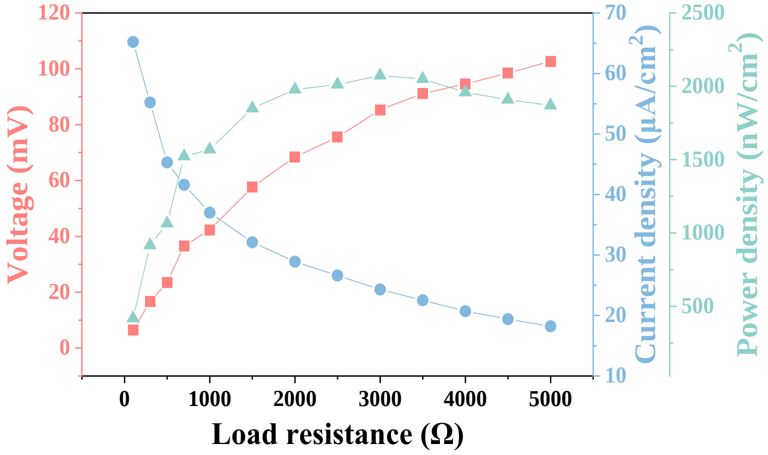

Figure S11. Power density of the ionic-diode device with different load at a pressure stimulation of 50 kPa

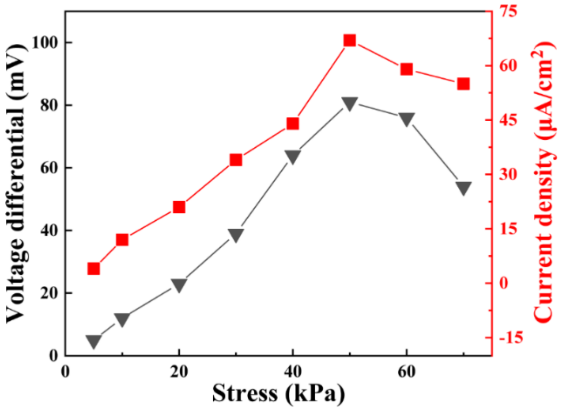

Figure S12. Effect of pressure on the potential of PSSNa/PDACl component.

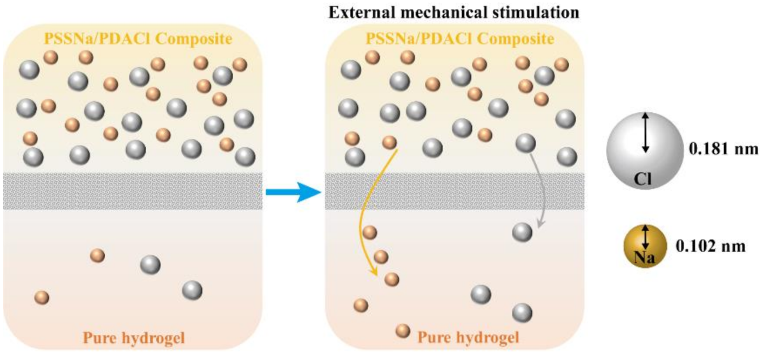

Figure S13. a) Diagram of ion transport in AB/0 model with and without external force.

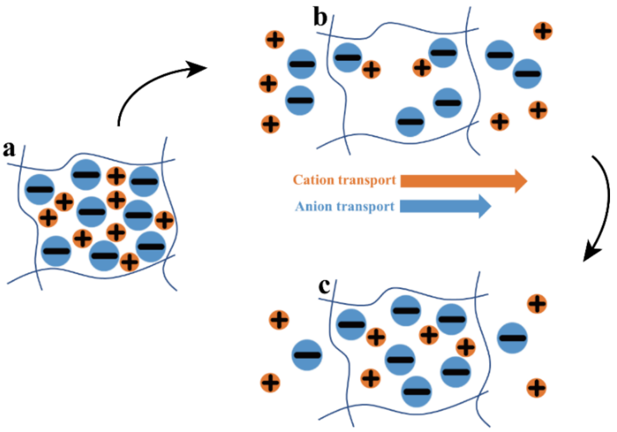

Figure S14. a) Polymer gels containing large amounts of anions and smaller mobile cations. b) Electrolyte flow outward from the compression region entrains the ionic species at different rates, generating charge separation. c) Ion separation is reduced when flow slows, due to back diffusion and electrophoresis.

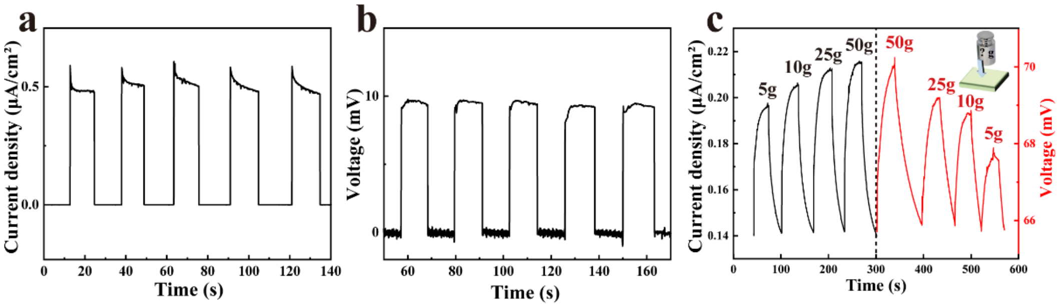

Figure S15. Performance of AB/0 model. a) Current and (b) potential voltage signal diagrams of AB/0 model (50 kPa). c) Changes of current and potential voltage under external force stimulation

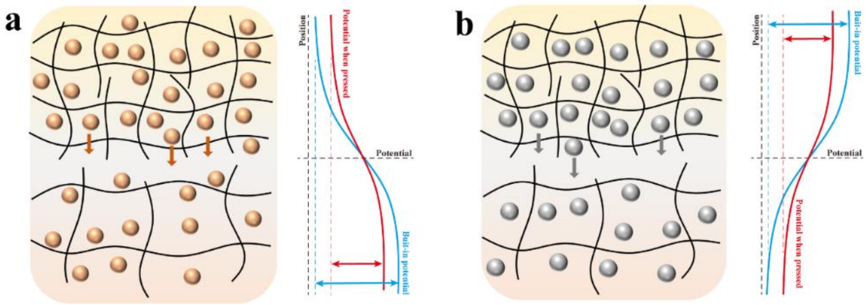

Figure S16. Diagram of ion transport in A_1/A_2 model with (a) sodium ion and (b) chloride ion.

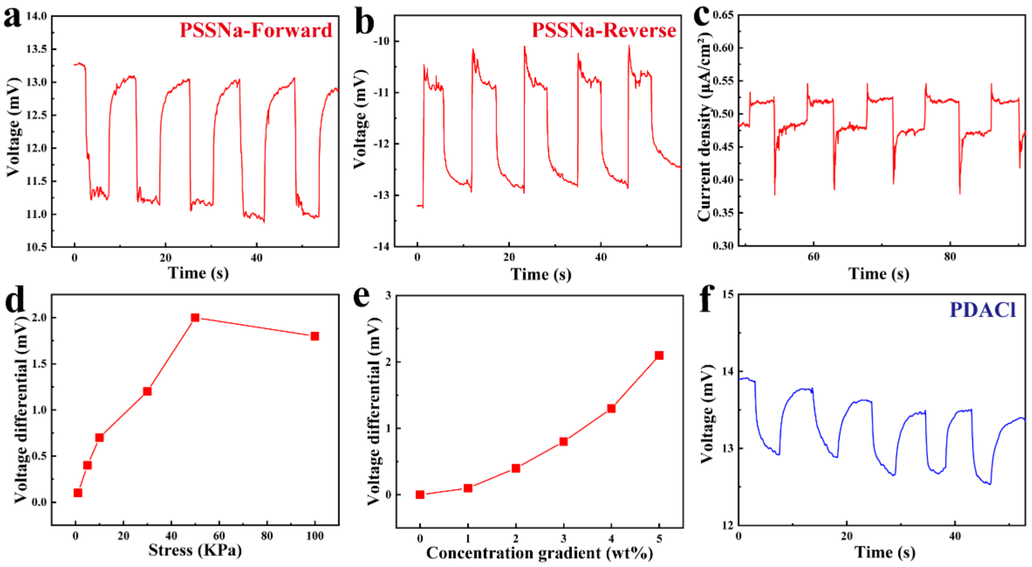

Figure S17. Performance of A_1/A_2 model device. a) Forward and (b) reverse potential voltage signal diagrams and c) Current signal diagram of PSSNa/PSSNa component (50 kPa). Effect of (d) pressure and (e) concentration gradient on potential voltage of PSSNa/PSSNa component. f) Potential voltage signal diagram of PDACl/PDACl component.

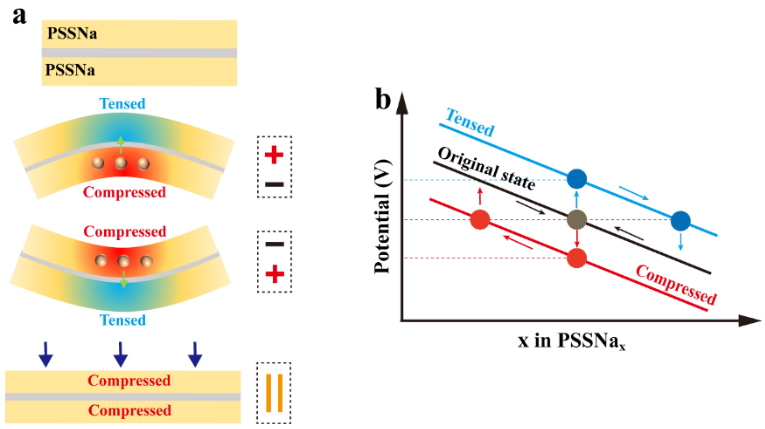

Figure S18. a) Schematic diagram of ion transport mechanism in A/A bending model. b) Thermodynamic perspective of the bending-unbending cycle

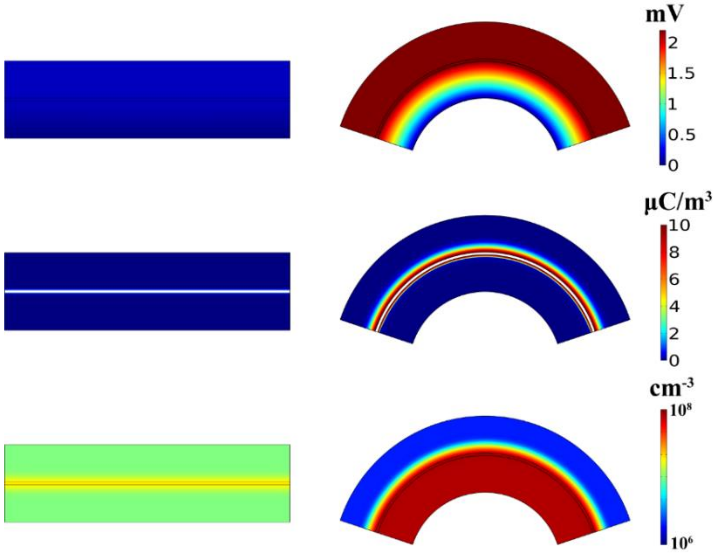

Figure S19. Simulation of potential gradient (top), space charge density (mid) and concentration distribution (bottom) of A/A model in bending deformation.

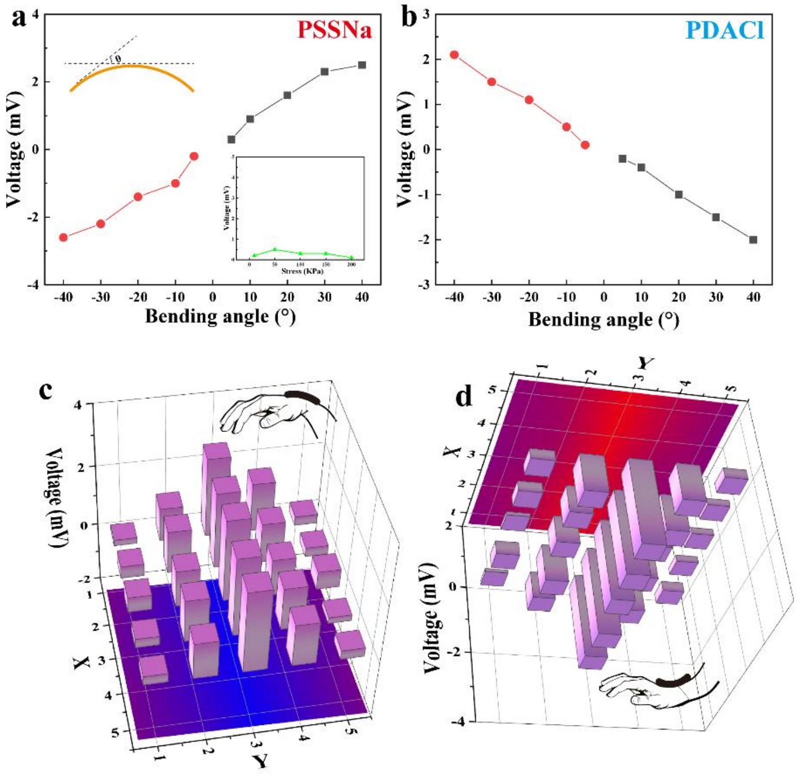

Figure S20. Performance of A/A model. Potential voltage trend of (a) PSSNa and (b) PDACl component device affected by bending angle (inset: Performance output under pressing stimulation). c,d) Array sensing detection wrist up and down bending signal diagram.

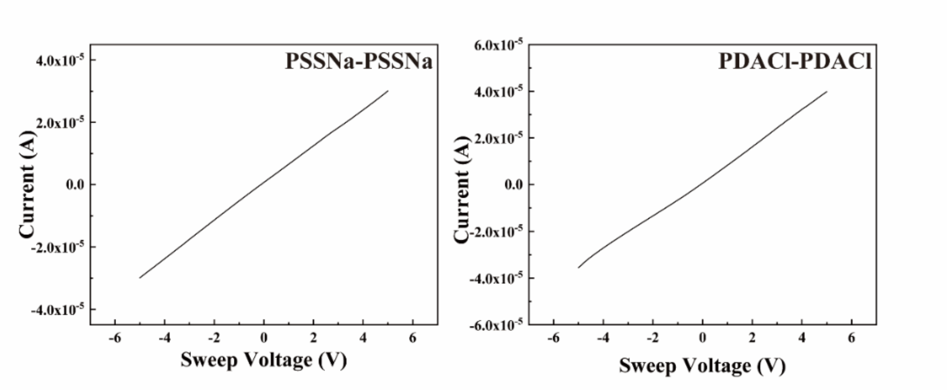

Figure S21. Voltage-current curves of ionic-diode devices with homogenous hydrogel nanocomposites.

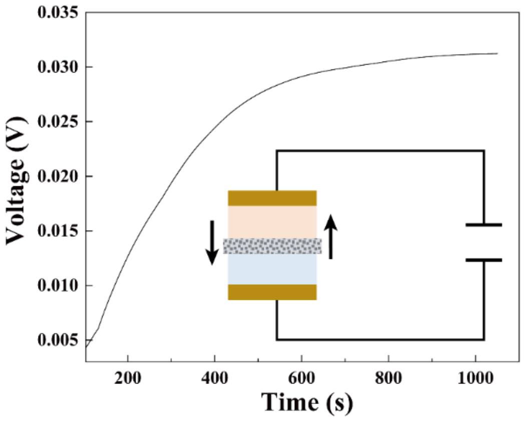

Figure S22. Potential signal of the ionic-diode device charging capacitor under external stimulation.

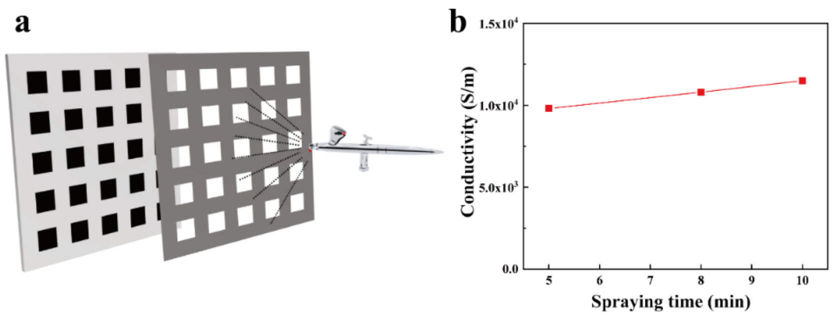

Figure S23. a) Schematic diagram of MXene electrode sprayed on PET substrate. b) Conductivity of the electrode after spraying.

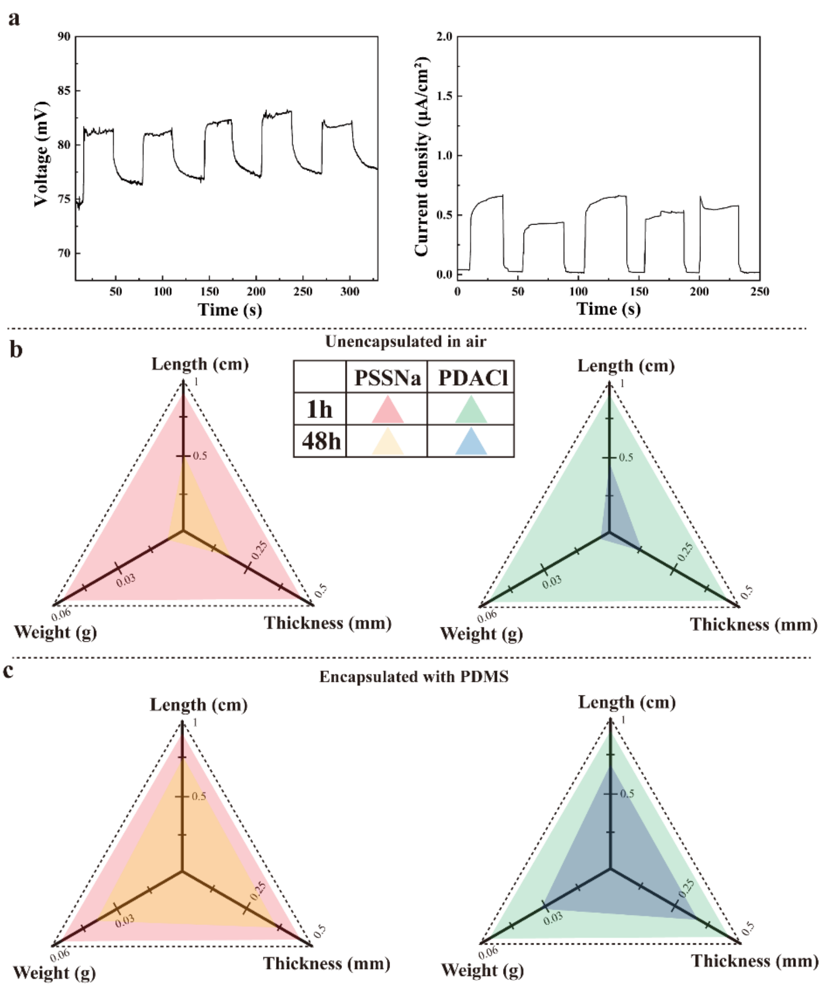

Figure S24. a) Current and voltage signal diagrams of A/B component after natural drying for 48 h without packaging（50kPa）. Changes in physical properties of PSSNa and PDACl composite hydrogel films exposed to air for 1 hour and 48 hours (b) without encapsulation and (c) encapsulated with PDMS.
